# Hypolipidemic, antioxidant and anti-atherosclerogenic effect of aqueous extract leaves of C*assia. occidentalis* Linn (Caesalpiniaceae) in diet-induced hypercholesterolemic rats

**DOI:** 10.1186/s12906-017-1566-x

**Published:** 2017-01-25

**Authors:** Ntchapda Fidèle, Barama Joseph, Talla Emmanuel, Dimo Théophile

**Affiliations:** 1 Department of Biological Sciences, Faculty of Science, University of Ngaoundéré, P.O. Box 454, Ngaoundéré, Cameroon; 2 Department of Chemistry, Faculty of Science, University of Ngaoundéré, P.O. Box 454, Ngaoundéré, Cameroon; 3 Department of Animal Biology and Physiology, Faculty of Science, University of Yaoundé 1, P.O. Box 812, Yaoundé, Cameroon

**Keywords:** *Cassia occidentalis*, Hypolipidemic, Anti-atherosclerotic, Lipid peroxidation TBARs

## Abstract

**Background:**

Hyperlipidemia and oxidative stress are major risk factors for atherosclerosis, and all three are among the most important risk factors for cardiovascular diseases. *Cassia occidentalis* aqueous extract has been used in African traditional medicine for the treatment of hypertension and associated cardiovascular diseases. This study was undertaken to evaluate the hypolipidemic and anti-atherosclerotic properties of the aqueous extract of the leaves of *C. occidentalis* in rats with hypercholesterolemia (HC).

Sixty Normocholesterolemic (NC) male rats were divided into six groups (*n* = 10) and fed a high-cholesterol (HC) diet for 30 days (5 groups), or normal rat chow (normal control group). The plant extract was administered to animals at the increasing dose of 240, 320 and 400 mg/kg. After 4 weeks of treatment 5 rats out of 10 were sacrificed, blood samples, aorta, liver, and fresh faecal were collected and processed for biochemical tests. The experiments were conducted under the same conditions with a group of rat treated with Atorvastatin (1 mg/kg), used positive control. The effects of *C. occidentalis* on weight gain, water and food consumptions, levels of serum lipids and lipoprotein lipid oxidation and stress markers in blood and liver were also examined.

**Results:**

A significant body weight gain was observed in general in all the group of animals without any treatment after 4 weeks. During the treatment period, the *C. occidentalis* extract induced a significant increase (*P* < 0.01) in water consumption and food intakes. After 4 weeks of treatment with hypercholesterolemia, the body temperature and organ weights including the liver, kidney, heart and the testis did not present any significant change. The administration of *C. occidentalis* extract significantly (*p* < 0.05) prevented the elevation in TC, LDL-C, VLDL-C, hepatic and aortic TG and TC. The atherogenic, triglycerides, and lipid peroxidation (TBARS) index were also decreased in the rats treated with the plant extract. *C. occidentalis* favoured the performance of faecal cholesterol. It also significantly inhibited the changes and the formation of aortic atherosclerotic plaques.

**Conclusion:**

This study provides evidence of hypolipidemic and antiatherosclerotic effects of *C. occidentalis* extract. *C. occidenntalis* aqueous extract reduced bad cholesterols, triglycerides and increasing good cholesterols in rats subjected to a feeding regime enriched with cholesterol. The results support the traditional use of the extract of this plant in the treatment of hypertension and diabetes.

## Background

Hyperlipidemia and oxidative stress are major risk factors for atherosclerosis, and all three are among the most important risk factors for cardiovascular diseases and conditions [1, 2]. The cardiovascular diseases constitute one of the absolutely largest public health problem in the world. According to the World Health Organization statistics [3], they are responsible of more than 17 million deaths annually. The cardiovascular diseases are associated to several cardio-metabolic risk factors such as hypercholesterolemia, diabetes, high blood pressure, obesity and sedentarity [4]. Dyslipidemia is a very frequent metabolic disorder which is characterized by an increase of the rates of triglycerides (TG), total cholesterol (CT), cholesterol of the low density lipoprotein (LDL-c) and a reduction of the cholesterol high density lipoprotein (HDL-c) [4]. A huge body of population based and experimental evidence shows that high levels of plasma low density lipoprotein (LDL-c) cholesterol and total cholesterol considerably increase the risk for developing atherosclerosis and associated arterial hypertension [5, 6]. Other changes in lipid parameters associated with atherosclerosis include decreases in high density lipoprotein (HDL-c) cholesterol and increases in triglycerides. It is well documented that hypercholesterolemia contributes to the development of the atherosclerosis, with hypertension and the renal failure [7]. The assumption of responsibility of the hypercholesterolemia in reduction of mortality as well as which has occurred of the events cardio/neurovasculaires, this via the reduction in the blood concentration of cholesterol related to the lipoproteins of low density (LDL-c). The low-density lipoprotein cholesterol (LDL-c) reduction is correlated with the magnitude of cardiovascular risks reduction.

For many decades medicinal plants have been used to prevent or treat various diseases. They are used throughout the world, for their hypoglycemia, hypolipidemia or antioxidant activities [8, 9]. *Cassia occidentalis* Linn. (Caesalpiniaceae) is a diffuse shrub (usually annual), with loosely spreading branches (60–150 cm long), and can grow up to an altitude of 1500 m [10]. Different parts of this plant have been reported to possess anti-inflammatory, antihepatotoxic [11], antibacterial [12], antiplasmodial [13] and antidiabetic [14] activities. They possess purgative, tonic, febrifugal, expectorant, and diuretic properties. The plant is also used to cure sore eyes, hematuria, rheumatism, typhoid, asthma, hemoglobin disorders and it is also reported to cure leprosy. A wide range of chemical constituents isolated from *C. occidentalis* include sennoside, anthraquinone glycoside [15], fatty oils, flavonoid glycosides, galactomannan, polysaccharides, and tannins [16]. Although leaves aqueous extract of *C. occidentalis* were reported to possess diuretic effects [17], no data on the effect of this medicinal plant on cardiovascular diseases and conditions are available. The present study therefore aimed at evaluating the anti-dyslipidemic, antioxidant, and anti-atherogenic effects of *C. occidentalis* leaf aqueous extracts and potential mechanisms driving its putative protective and therapeutic effects.

## Methods

### Plant material

Fresh leaves of *C. occidentalis* used in this study were harvested in Mora 60 Km from Maroua, the largest city in the Far North Region, Cameroon in July 2013. They were identified by experts of the National Herbarium of Cameroon and a sample was deposited (specimen N^0^ 21057/SFR/CAM). Leaves of *C. occidentalis* were extracted as described previously [17].

### Preparation of leave aqueous extract

Fresh leaves of *C. occidentalis* were soaked in distilled water (1000 g for 1 L at room temperature) for 12 h. The macerate was filtered through Whatman filter paper N^o^ 3, and the filtrate concentrated in a rotary evaporator at 40 °C for 24 h. This process was repeated until an oily paste extract was obtained (130 g), which represented the concentrated crude extract of *C. occidentalis* leaves. The extract was stored at −20 °C until use. The solution of *C. occidentalis* extract with the highest concentration tested was prepared by dissolving 800 mg of the concentrated crude extract obtained previously in 10 ml of distilled water (80 mg/mL concentration). The other solutions used in the study were 4:5, 3:5, 2:5, and 1:5 dilutions of this solution in distilled water. Solutions were given *per os* in a volume of 5 ml/kg body weight, thus, the increasing doses of aqueous extract of *C. occidentalis* tested were 80, 160, 240, 320, and 400 mg/kg.

### Preliminary qualitative phytochemical analysis

In order to identify the chemical structure of the compounds responsible for the antioxidant and anti-atherosclerogenic activity, preliminary tests of the phytochemical study were conducted following the procedures described by Trease and Evans [18]. Briefly, Essential oils from the aqueous extract of *C. occidentalis* were extracted with hexane. These extracts were then stitched onto plates of thin layer chromatography on silica, the first disclosure was obtained by ultraviolet radiation (254 nm and 365 nm) and then with vanillin. Analytical tests for the identification of different families of metabolites in crude extracts of the leaves were performed at the national Institute of Medicinal Plants for Medicinal research (IMPM, Cameroon).

### Animals

Sixty normo-cholesterolemic (NC) male Wistar rats (178.35 ± 1.46 g) were purchased from Yaounde (Cameroon) Pasteur Institute and acclimated to the Laboratory of Medicinal Plants, Health and Galenic Formulation of the Department of Biological Sciences, University of Ngaoundere (Cameroon). Animals were housed under controlled room temperature (24 ± 2 °C) and had ad libitum access to food [National Veterinary Laboratory (LANAVET), Garoua, Cameroon] and tap water. Animals were monitored for signs of general toxicity, under the supervision of a veterinarian. The number of animal per group approved in the experiments by the institutional committee of ethics was five. All experimental procedures were approved by the institutional Ethical committee of Department of Biological Science of the university of Ngaoundéré (ECDBSUN 15/01/2015/UN/FS/DSB).

### Experimental procedures

Normo-cholesterolemic (NC) (60 rats) were divided into 6 groups of 10 rats. Five groups were fed for 4 weeks with a diet consisting of 50% Corn Starch, 11.25% Rice Powder, 01% vegetable oil, 10% egg white, 08% fish meal, 19% Cellulose, 0.125% mineral complex, 0.125% vitamin Complex and 0.50% Salt [19, 20]. For induction of hypercholesterolemia (HC), 1% of cholesterol was added in the feed of rats. The nutrient contents of the NC (g/100 g food) diet were: total lipid (19.70 ± 0.28); protein (32.95 ± 2.4); ash (0.02 ± 0.005); fiber (12.33 ± 1.50); carbohydrates (35 ± 2.3) [21]. The plant extract was administered to animals at the increasing dose of 240, 320 and 400 mg/kg. After 4 weeks of treatment 5 rats out of 10 were sacrificed, blood samples, aorta, liver, and fresh faecal were collected and processed for biochemical tests. The remaining 5 rats were sacrificed 4 weeks after the end treatment and blood were collected again for biochemical analysis. Results were later compared to first group to confirm the anti-atherogenic properties of the leaves extract. Blood collected in heparinized tubes, were centrifuged at 3000 rev/min for 10 min; the supernatant (plasma) was used for the enzymatic determination of total cholesterol, HDL-c and triglycerides and malondialdehyde. Blood pellet was used in the preparation of hemolysates while the portion of the liver collected was used to prepare liver homogenates for the dosage of catalase, hydroperoxides and proteins. The experiments were conducted under the same conditions with Atorvastatin^®^ (1 mg/kg), as pharmacological reference substance.

### Body temperature monitoring

Body temperature of treated rats was monitored daily 5 h after treatment using a rat rectal thermometer. It was inserted at a distance of approximately 2 mm in the anus.

### Statistical analysis

Data obtained from the different experimental groups were compared by one-way ANOVA followed by LSD test for post hoc analysis, using Origin software (Origin Lab, Northampton, MA, USA). Test groups were compared to normal, disease, and positive control groups. Differences with *P* < 0.05 were considered significant. Data are presented as mean ± SEM.

## Results

### Body weight

**Fig. 1:**
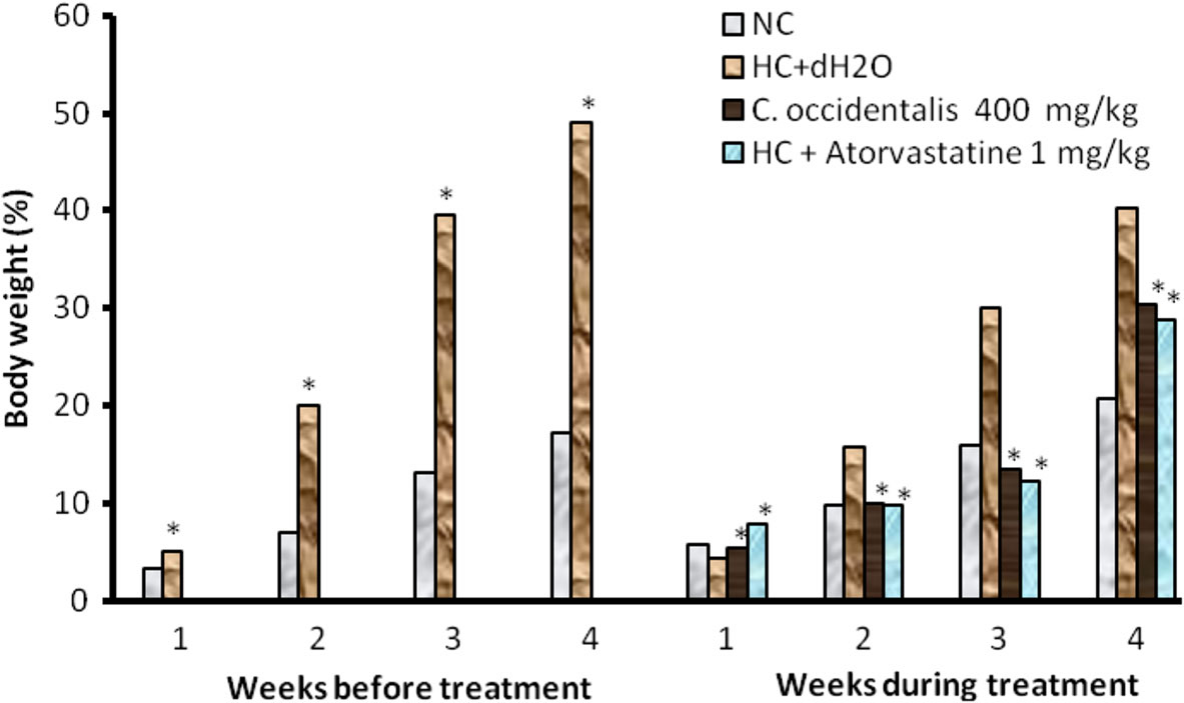
variation of the body weight of the rats per week. Values are represented as mean ± standard error of mean, *n* = 5. dH2O: distilled water. HC: Hypercholesterolemic rats; NC: Normocholesterolemic rats

During the 4 weeks period of induction, the body weight of animals in the Normocholesterolemic fed with cholesterol-free diet, significantly increased (*P* < 0.05) from 3.22% in week one to 17.12% at week 4. Animals fed with a diet rich in cholesterol, have seen their body weights increased significantly (*P* < 0.05) to 5.11% in week 1 to 49.11% at week 4 (Fig. 1). During the four weeks of treatment extract-treated animals showed a dose-dependent loss of body weight. Animals treated daily with a dose of 240 mg/kg of the extract experienced a decreased in the relative body weight of 2.67% and 8.39% respectively in week 1 and week 4. with the slope *y* = −3.14*x* + 264.6, *r* ^2^ = 0.97. At a daily dose of 320 mg/kg *y* = −5.67*x* + 264.2, *r* ^2^ = 0.95, and animals that received the extract at a dose 400 mg/kg, have seen their body weight decreased from 268.33 ± 0.82 g/rat to 247.66 ± 3.68 g/rat in week 1 and from 247.66 ± 3.68 g/rat to 199.77 ± 3.87 g/rat in week 4, leading to a decrease of 8.34% and 34.31% respectively with the slope *y* = −9.83*x* + 271.1, *r* ^2^ = 0.98 (Fig. 1). The increase in body weight observed in animals receiving Atorvastatin was also significant (*P* < 0.05) (Fig. 1). A significant body weight gain was observed in general in all the group of animals without any plant extract treatment 4 weeks after (Fig. 1).

### Water consumption and food intake

Average daily water and food intake are shown in Tables 1 and 2. During the treatment, the *C. occidentalis* extract caused a significant increase (*P* < 0.01) in water consumption and food intake. At a dose of 400 mg/kg, the increase of drinking water was 29,89% and 49,88% respectively in the first week and fourth week. Four weeks after treatment, water consumption decreased in animals previously treated with aqueous extract. At the dose of 400 mg/kg, water consumption decreased by 60.61% and 180.77% respectively in week 1 and week 4, when compared to the water consumption during the treatment at the same dose (Table 1). On the other hand, food intake also increased comparably in all groups. HC + distilled water group showed an increase of 44.66 ± 1.12% and 57.87 ± 1.23% respectively in the 1st week and in the fourth week in food consumption during treatment (*y* = 1.25*x* + 17.4, *r* ^2^ = 0.99) (Table 2). During the 4 weeks of hypercholesterolemia treatment, food consumption significantly decreased (*P* < 0.05) in treated animals compared to HC + distilled water. At the dose of 400 mg/kg, there was a decrease in food intake of 39.55 ± 1.33% in the first week and 23.68 ± 1.45% in week 4 compared to HC + H_2_0 (*y* = 2.24*x* + 16.76, *r* ^2^ = 0.97). At a dose of 240 mg/kg (*y* = 3.45*x* + 13.56, *r* ^2^ = 0.99), 320 mg/kg (*y* = 2.32*x* + 18.83, *r* ^2^ = 0.99), and Atorvastatin (*y* = 1.69*x* + 19.14, *r* ^2^ = 0.93)] (Table 2).

**Table 1:** Variation of water consumption

	Drugs	Doses mg/kg	1st week (ml/rat/week)	2nd week (ml/rat/week)	3rd week (ml/rat/week)	4th week (ml/rat/week)
	HC + Distilled water	**0**	116.12 ± 1.21	137.12 ± 1.12	142.10 ± 1.22	146.21 ± 1.32
During treatment	HC + Extract	**240**	179.04 ± 1.23*^a^	158.32 ± 1.16*^a^	160.24 ± 1.14*^a^	179.31 ± 1.22*^a^
		**320**	139.43 ± 1.22**^a^	200.31 ± 1.32**^a^	215.12 ± 1.32**^a^	236.83 ± 1.11**^a^
		**400**	178.43 ± 2.33**^a^	239.25 ± 1.67**^a^	265.27 ± 2.37**^a^	289.66 ± 1.23**^a^
	HC + Atorvastatin	**1**	145.95 ± 1.12	164.75 ± 1.13	174.15 ± 1.13	166.64 ± 1.12
After treatment	HC + Extract	**240**	86.33 ± 1.17**^b^	88.44 ± 1.17**^b^	89.22 ± 1.17**^b^	89.10 ± .1.20**^b^
		**320**	95.32 ± 1.66**^b^	86.22 ± 1.16**^b^	97.12 ± 1.77**^b^	98.44 ± 1.25**^b^
		**400**	103.93 ± 1.11**^b^	99.77 ± 1.11**^b^	100.93 ± 1.01**^b^	101.65 ± 0.83**^b^
	HC + Atorvastatin	**1**	141.85 ± 3.72	162.75 ± 2.76	160.75 ± 3.78	166.69 ± 1.03

Values are means ± S.E.M., *n* = 5, *P < 0.05, **P < 0.01 significant difference compared to HC+ Distilled water. *HC* Hypercholesterolemic rat; ^a^significant difference compared to HC+ Distilled water; ^b^significant difference compared to animals previously treated with aqueous extract during the treatment at the same dose

**Table 2:** Variation of the food intake during treatment

	Drugs	Doses mg/kg	1st week (%/week)	2nd week (%/week)	3rd week (%/week)	4th week (%/week)
	HC + Distilled water	**0**	44.66 ± 1.12%	54.12 ± 1.10%	55.11 ± 1.22%	57.87 ± 1.23%
During treatment	HC + Extract	**240**	56.80 ± 1.12%	54.47 ± 1.14%	50.23 ± 1.24%*^a^	47.47 ± 1.32%**^a^
		**320**	54.23 ± 1.45%	52.39 ± 1.09%*^a^	50.22 ± 1.19%*^a^	45.39 ± 1.23%**^a^
		**400**	39.55 ± 1.33%*^a^	35.54 ± 1.32%*^a^	28.34 ± 1.12%**^a^	23.68 ± 1.45%**^a^
	HC + Atorvastatin	**1**	36.17 ± 1.32%**^a^	37.16 ± 1.06%**^a^	39.37 ± 1.32%**^a^	43.41 ± 1.13%**^a^
After treatment	HC + Extract	**240**	53.13 ± 1.56%	56.32 ± 1.21%*^b^	58.87 ± 1.22%*^b^	62.23 ± 1.12%**^b^
		**320**	56.16 ± 1.32%*^b^	55.33 ± 1.41%*^b^	60.77 ± 1.42%*^b^	62.44 ± 1.54%**^b^
		**400**	47.33 ± 1.22%*^b^	49.33 ± 1.25%**^b^	52.22 ± 1.32%**^b^	59.80 ± 1.11%**^b^
	HC + Atorvastatin	**1**	39.21 ± 1.14%*^b^	41.89 ± 1.31%*^b^	45.82 ± 1.34%*^b^	49.15 ± 1.22%**^b^

Values are means ± S.E.M., *n* = 5, *P < 0.05, **P < 0.01, *HC* Hypercholesterolemic rat; ^a^significant difference compared to HC+ Distilled water; ^b^significant difference compared to animals previously treated with aqueous extract during the treatment at the same dose

### Organ weight

After 4 weeks of treatment of hypercholesterolemia, the Organ weight including the liver, kidney, heart and the testis of animals received extract at doses (240, 320 and 400 mg/kg) compared with the group hypercholesterolemic untreated and groups normocholesterolemic did not present any significant change ( < 0.05) (Table 3).

**Table 3:** Effects of *C. occidentalis* extract administration for 4 weeks on organ weight

Group		Organ weight			
		Liver	Heart	Kidney	Testis
HC + dH_2_O		3.04 ± 0.71	0.34 ± 0.07	0.66 ± 0.35	0.55 ± 0.12
C. occidentalis treated	240 mg/kg	3.04 ± 0.15	0.33 ± 0.03	0.64 ± 0.07	0.53 ± 0.17
	320 mg/kg	3.05 ± 0.91	0.32 ± 0.07	0.62 ± 0.53	0.53 ± 0.14
	400 mg/kg	3.01 ± 0.25	0.32 ± 0.04	0.61 ± 0.04	0.53 ± 0.13
Atorvastatin (1 mg/kg)		3.02 ± 0.18	0.32 ± 0.07	0.61 ± 0.06	0.52 ± 0.16
NC		3.01 ± 0.26	0.30 ± 0.10	0.61 ± 0.25	0.52 ± 0.18

Values are represented as mean ± standard error of mean, *n* = 5. No significant change was observed. *dH2O* distilled water, *HC* Hypercholesterolemic rats, *NC* Normocholesterolemic rats

### Body temperature

No significant change was observed in the body temperature in treated animals compared to Hypercholesterolemic + distilled water and atorvastatin; or after inter-group comparisons, during the treatment period (Table 4) and 4 weeks after (data not shown). Effects of aqueous extract leaves of *C. occidentalis* on body temperature measured daily 5-h after treatment are presented in Table 4.

**Table 4:** Effect of *C. occidentalis* extract administration for 4 weeks on body temperature

Time (days)	Extract of *C. occidentalis* (mg/kg)				Atorvastatin (1 mg/kg)
	Control	240	320	400	
1	36.11 ± 0.13	36.12 ± 0.12	36.13 ± 0.11	37.13 ± 0.13	36.13 ± 0.13
2	36.12 ± 0.12	36.13 ± 0.15	37.14 ± 0.13	36.11 ± 0.14	36.12 ± 0.13
3	37.13 ± 0.12	36.13 ± 0.11	35.15 ± 0.11	36.13 ± 0.13	37.14 ± 0.11
4	36.12 ± 0.15	36.14 ± 0.12	37.12 ± 0.12	36.12 ± 0.12	36.11 ± 0.13
5	36.13 ± 0.11	36.11 ± 0.13	36.14 ± 0.13	36.12 ± 0.14	36.12 ± 0.11
6	36.14 ± 0.13	36.14 ± 0.13	36.12 ± 0.12	36.13 ± 0.11	36.13 ± 0.11
7	36.11 ± 0.12	36.12 ± 0.14	36.13 ± 0.13	37.12 ± 0.13	36.13 ± 0.13
30	37.09 ± 0.14	36.14 ± 0.12	36.14 ± 0.14	36.12 ± 0.11	36.12 ± 0.14

Values are represented as mean ± standard error of mean, *n* = 5. No significant change was observed

*dH2O* distilled water

### Effect of aqueous extract of *C. occidentalis* on blood lipid parameters

#### During treatment

Rat treated with extract showed a significant reduction and dose-dependent of TC level. TC decreased from 156.09 ± 0.92 mg/dl to 135.51 ± 0.51 mg/dl, at a dose of 240 mg/kg, 116.63 ± 0.54 mg/dl at a dose of 320 mg/kg, and 106.07 ± 0.69 mg/dl at a dose of 400 mg/kg, leading to a decrease of 13.18%, 25.28% and 32.05% respectively. TC also decreased in the group which received a treatment compared to the normocholesterolemic (Table 5).

**Table 5:** Effect of aqueous extract of *C. occidentalis* on blood lipid parameters

Experimental groups		Lipid parameters (mg/dL)						
		TC	TG	VLDL-c	HDL-c	LDL-c	LDL/HDL	CT/HDL
HC rats + dH_2_O		156.09 ± 0.92	141.01 ± 0.63	28.21 ± 0.73	31.05 ± 0.48*	109.28 ± 0.49	5.87 ± 0.05	8.44 ± 0.82
HC +4 weeks treatment	240 mg/kg	135.51 ± 0.51*	101.62 ± 0.98*	20.33 ± 0.79*	26.73 ± 0.47	81.53 ± 0.5*	3.05 ± 0.07*	6.25 ± 0.34*
	320 mg/kg	116.63 ± 0,54*	95.06 ± 0.57*	19.02 ± 0.66*	28.35 ± 0.58*	53.02 ± 0.83*	1.87 ± 0.06*	4.59 ± 0.16*
	400 mg/kg	106.07 ± 0.69*	85.15 ± 0.64*	16.65 ± 0.38*	34.77 ± 0.6*	36.17 ± 0.34*	0.96 ± 0.04*	3.96 ± 0.15*
HC 4 weeks after treatment	240 mg/kg	130.12 ± 0.11*	98.13 ± 0.30*	18.30 ± 0.70*	27.12 ± 0.17	80.31 ± 0.15*	2.96 ± 0.02*	4.79 ± 0.13*
	320 mg/kg	110.22 ± 0,34*	89.16 ± 0.33*	16.12 ± 0.25*	30.22 ± 0.32*	48.22 ± 0.23*	1.59 ± 0.03*	3.64 ± 0.11*
	400 mg/kg	99.14 ± 0.44*	79.17 ± 0.24*	14.25 ± 0.31*	36.43 ± 0.21*	33.27 ± 0.14*	0.91 ± 0.02*	2.72 ± 0.12*
HC + Atorvastatin	1 mg/kg	95.23 ± 0.57*	86.84 ± 0.84*	17.27 ± 0.63*	31.05 ± 0.48*	39.06 ± 0.97*	1.26 ± 0.04*	3.29 ± 0.42*
NC rats		69.61 ± 0.92	69.46 ± 0.58	14.39 ± 0.57	30.83 ± 0.39	25.38 ± 0.66	0.83 ± 0.07	2.28 ± 0.07

Data are represented as mean ± standard error of mean, *n* = 5. One-way ANOVA + LSD test against hypercholesterolemic (HC) rats: *P < 0.05. *dH*_*2*_ *O* distilled water, *HDL-c* high density lipoprotein cholesterol, *LDL-c* low density lipoprotein cholesterol, *NC* normocholesterolemic, *T* treatment, *TC* total cholesterol, *TG* triglyceride, *VLDL-c* very low density lipoprotein cholesterol

TG decreased from from 141.01 ± 0.63 mg/dl to 101.62 ± 0.98 mg/dl leading to a decrease of 27.93%, 32.58% and 39.62% respectively at a doses 240, 320 and 400 mg/kg compared to the normocholesterolemic (Table 5).

Table 4 also shows HDL-c rate during treatment. Rats treated with Atorvastatin showed a significant decreased of HDL-c level compared to the normocholesterolemic, leading to a decrease of 3.33%. Rat treated with extract showed a significant reduction and dose-dependent of HDL-c levels, compared to the normocholesterolemic, leading to a decrease of 13.33%, 6.66% and an increase of 13.33% respectively at the doses of 240, 320 and 400 mg/kg compared to the normocholesterolemic (Table 5).

LDL-c rate decrease from 109.28 ± 0.49 mg/dL with the normocholesterolemic to 53.02 ± 0.83 mg/dL for the rats treated with Atorvastatin, and to 81.53 ± 0.5 mg/dL, 53.02 ± 0.83 mg/dL, 36.17 ± 0.34 mg/dL for the rats treated with extract at the doses of 240, 320 and 400 mg/kg respectively compared to the normocholesterolemic (Table 5).

VLDL-c levels decrease from 28.21 ± 0.73 with the normocholesterolemic to 20.33 ± 0.79, 19.02 ± 0.66 and 16.65 ± 0.38, with the rats treated with extract with the doses of 240, 320 and 400 mg/kg respectively compared to the normocholesterolemic. Leading to a decrease of 27.93%, 32.57% and of 40.97% respectively at the doses of 240, 320 and 400 mg/kg compared to the normocholesterolemic (Table 5). Treatment with Atorvastatin, showed a decrease of 38.78%.

#### Ratio of LDL/HDL-C and TC/HDL-c

In normocholesterolemic, the TC/HDL-c had a non-significant increase (*P* > 0.05), whereas LDL/HDL-c also showed no significant differences (*P* > 0.05). Hypercholesterolemic rat treated with Atorvastatin showed a 365.87% reduction in LDL/HDL-c against 156.53% in TC/HDL-c levels. Rats treated with the extract at the dose of 400 mg/kg showed a reduction of LDL/HDL-c ratio of 511.45% when compared to the Hypercholesterolemic + distilled water rats. This reduction was also observed in the TC/HDL-c and the value decreased from 8.44 ± 0.82 in Hypercholesterolemic + distilled water rats to 3.96 ± 0.15 in rats treated with the extract at dose of 400 mg/kg, equivalent to a reduction of 113.13%. (Table 5). LDL/HDL-c ratio decreased in a dose-dependent manner when compared with Hypercholesterolemic + distilled water rats.

#### After treatment

The interest of this study consisted in checking that after 4 weeks of treatment, the extract would always act.the aim would be to highlight vasodilators which can reconstitute the level of integrity of the endothelium and the production and the diffusion of the oxide nitrite. Four weeks after replacement of Hypercholesterolemic diet with normal rat chow and concomitant end of treatments, physiological levels of blood lipid parameters were still observed in animals treated with either Atorvastatin or a dose of extract (Table 5). Compared to those given normal rat chow (normocholesterolemic rats), animals given a diet enriched in cholesterol 4 weeks and only distilled water (Hypercholesterolemic rats) showed significant (*P* < 0.05) increases in total cholesterol (124.23%), in triglycerides (103.01%), in very low density lipoprotein (VLDL-c) cholesterol (96.03%), in LDL cholesterol (330.57%), and a decrease in HDL cholesterol (3.26%). These alterations were prevented in Hypercholesterolemic rats treated with Atorvastatin, as expected, but also by the three doses of extract in a dose-dependent fashion (Table 5).

#### Ratio of LDL/HDL-c and TC/HDL-c

There were a significant changes in LDL/HDL-c and TC/HDL-c ratios in rats, 4 weeks after treatment. LDL/HDL-c ratio decreased from 5.87 ± 0.05 in Hypercholesterolemic + distilled water rats without diet consisting to 0.91 ± 0.02 in the rats treated with *C. occidentalis* at the dose of 400 mg/kg after treatment. The TC/HDL-c also decreased from 8.44 ± 0.82in Hypercholesterolemic + distilled water rats without diet consisting to 2.72 ± 0.12 in the rats treated with *C. occidentalis* at the dose of 400 mg/kg after treatment. (Table 7). The of *LDL/HDL-c* ratios and *TC/HDL-c* ratios were significantly increased in Hypercholesterolemic rats (*P* < 0.05) compared to normocholesterolemic rats (Table 4). Such alteration in these ratios was prevented in Hypercholesterolemic rats by atorvastatin treatment, but also by treatments with extracts of *C. occidentalis*, in a dose-dependent fashion (Table 5).

### Effect of the aqueous extract of *C occidentalis* on oxidative stress markers in liver homogenates and blood

The effect of the aqueous extract of *C. occidentalis* on various markers of oxidative stress markers in liver homogenates, hemolysates, and plasma was dose-dependent as shown in (Table 6). Catalase activities in liver homogenates and in hemolysates were significantly decreased (*P* < 0.05). The extract also induced a significant decrease in hydroperoxide amount in liver homogenates (*P* < 0.001), and an increase in blood plasma (*P* < 0.05). Plasma and liver malondialdehyde amounts were significantly decreased (*p* < 0.05). Glutathione concentration in plasma was decreased (*P* < 0.01). Protein amounts were decreased in liver homogenates (*P* < 0.01) and increased in hemolysates (*P* < 0.05). Protein level was decreased in liver homogenates (*y* = −5.77*x* + 52.7, *r* ^2^ = 0.93) and increased in hemolysates (*y* = 3.42*x* + 44.8, *r* ^2^ = 0.95) of Hypercholesterolemic rats treated with the extract. Changes in liver homogenates were significant at higher doses of extract (*P* < 0.01), whereas Catalase level was increased in the liver (*y* = 0.04*x*−0.03, *r* ^2^ = 0.772) of Hypercholesterolemic rats treated with the extract (Table 6). Such increase was significant at the highest dose tested (*P* < 0.05). Catalase level was also increased in hemolysates of these animals (*y* = 0.05*x*−0.014, *r* ^2^ = 0.84). Malondialdehyde (MDA) level was decreased in the liver (*y* = −0.77*x* + 11.19, *r* ^2^ = 0.94) and in blood plasma (*y* = −3.58*x* + 33.4, *r* ^2^ = 0.96) of Hypercholesterolemic rats treated with the extract (Table 5). These decreased were significant at higher doses tested (*P* < 0.05). Hydroperoxide (ROOH) level was decreased in the liver at the highest dose tested (*y* = 0.02*x* + 0.03, *r* ^2^ = 0.98, *P* < 0.05), and in plasma (*y* = −0.19*x* + 1.7, *r* ^2^ = 0.80) (Table 6).

**Table 6:** Effect of the aqueous extract of *C. occidentalis* on markers of oxidative stress

	Experimental groups	MDA (μM/100 g of tissue)	ROOH (μM/100 g of tissue)	CAT (mMH2O2/min per g of protein)	Protein (g/100 g of tissue)	Glutathione (mmol/L)
Liver homogenates	dH_2_O	10.77 ± 1.44	1.28 ± 0.10	0.02 ± 0.13	54.11 ± 0.54	---
	400 mg/kg extract	8.89 ± 1.77*	0.87 ± 0.15***	0.19 ± 0.17*	29.33 ± 3.16**	---
Blood plasma	dH_2_O	19.67 ± 1.23	0.04 ± 0.11	---	---	0.44 ± 0,056
	400 mg/kg extract	7.13 ± 1.44***	0.07 ± 0.12*	---	---	0.23 ± 0,033**
Blood pellet hemolysates	dH_2_O	---	---	0.01 ± 0.014	43.77 ± 4.21	---
	400 mg/kg extract	---	---	0.14 ± 0.14***	52.65 ± 0.43*	---

*CAT* catalase, *MDA* malondialdehyde, *ROOH* hydroperoxide. Values are mean ± S.E.M., *n* = 5. ANOVA + LSD test vs. negative control: * *P* < 0.05, **P < 0.01, ***P < 0.001

### Effect of the aqueous extract of *C occidentalis* on bilirubin, fecal cholesterol and blood urea

*C. occidentalis* did not have any change on bilirubin elimination from blood. evertheless, it is necessary to note the lack of significant difference of bilirubin level between rats treated with aqueous extract and the untreated rats (Table 7). During the last 5 days of the treatment, faecal cholesterol was evaluated. Faecal cholesterol of rats treated with extract increased from 1.04 ± 0.27 mg/dl to 11.47 ± 0.75, 14.11 ± 1.63, 15.04 ± 1.98 mg/dl respectively at the doses of 240, 320, 400 mg/kg. Rats treated with atorvastatin showed an increase rate of fecal cholesterol of 1434.61% compared to the normocholesterolemic. Table 7 shows the blood urea level after treatment. It is deduced from this table that; there is no significant difference in the level of urea present in the blood of rats treated with aqueous extract and the untreated rats (Table 7).

**Table 7:** Effect of the aqueous extract of *C occidentalis* on bilirubin, Faecal cholesterol and blood urea

Experimental groups		Parameters (mg/dL)			
		Bilirubin	Urea	Creatinin	Faecal cholesterol
HC rats + dH_2_O		0,87 ± 0,18	0.47 ± 0.02	0.41 ± 0.02	5,58 ± 1,04
HC +4 weeks treatment	240 mg/kg	0,90 ± 0,12	0.31 ± 0.04*	0.26 ± 0.04*	11,47 ± 0,75*
	320 mg/kg	0,89 ± 0,14	0.28 ± 0.05*	0.24 ± 0.05*	14,11 ± 1,6*
	400 mg/kg	0,91 ± 0,15	0.26 ± 0.03*	0.22 ± 0.03*	15,04 ± 1,98*
HC + Atorvastatin	1 mg/kg	0,92 ± 0,11	0.27 ± 0.03	0.28 ± 0.03*	15,96 ± 1,64*
NC rats		0,94 ± 0,13	0.35 ± 0.03	0.25 ± 0.03	1,04 ± 0,27

Data are represented as mean ± standard error of mean, *n* = 5. One-way ANOVA + LSD test against dH_2_O: distilled rats: *P < 0.05

*HC* Hypercholesterolemic rats, *NC* Normo-cholesterolemic rats

### Phytochemical study

Phytochemical screening performed on crude extracts revealed the presence of several primary and secondary metabolites such as fatty acids, anthraquinones, glycosides, anthracenes, saponins, tannins, and coumarins. Phenolic compounds, triterpenes, volatile oils and sterols, but also flavonoids and alkaloids were also present in the extract. Thin layer chromatography (TLC) showed that the hexane extract and urine of treated rats contained four chemical fractions. These initial observations and findings suggest that the aqueous extract of leaves of *C. occidentalis* contains several chemical compounds with potential biological activities which deserve further investigation.

## Discussion

The aqueous extract of *C. occidentalis* reduced dose dependently and significantly the triglyceride and cholesterol levels in the rats. Ajayi *et al.* [22], reported that drugs with anticholesteremic properties are also antioxidant. This suggests aqueous extract of *C. occidentalis* may have antioxidant properties. This property could justify the use of the maceration of *C. occidentalis* in traditional medicine to treat hypertension and to reduce the triglyceride and cholesterol levels in blood [23, 24]. In our experiment, animal body weights were significantly increased compared to the controls. This increase could have resulted in the increase in the food and water intakes of the rats. Physiologically, the increase in the appetite could be due to orexine, the stimulative hormone of appetite (Balkan, 2002). Animals fed with a feeding diet rich in cholesterol, have seen their body weights increased significantly, thus developed obesity [25, 26]. This increase in body weight is due to the increase of fat tissue deposit much more on the level of the hip. We also noted that body weight in rat fed with HC + dH_2_O is significantly increasing in rat fed with NC before treatment in Fig. 1. This result means that the well nourished animals can take weight if they are in good health. But what we noted, it is with the difference of 1% of cholesterol giving in HC + dH_2_O, the Body weight in rat fed with HC + dH_2_O is significantly increasing than NC rat. We know that cholesterol induces the hormone synthesis such as cortisol, the aldosteron, the testosteron and the oestrogens which are the sex hormones may be the increasing in cholesterol in the HC + dH_2_O rats would have contributed to increase the rate of these hormones and induce significantly increasing of body weight in rat fed with HC + dH_2_O.

The treatment with the aqueous extract of *C. occidentalis*, in the rat fed with a diet rich in cholesterol and triglyceride compared with the untreated rat and normal rats, induced a reduction in the contents of VLDL-c. This reduction of cholesterol is found on the level of the TC, the LDL-c and Triglycerides. These results are similar with those of several works completed with other plant extracts, such as the aqueous extract of *Dunaliella salina* [27] and the ethanolic extract of *Crataegus pinnatifida* [28], in rats subjected to a feeding regime enriched with lipids. Considering that abnormal lipid profiles constituting the hallmark of HC-induced metabolic syndrome were also prevented in the liver by the extract concomitantly with a marked increase in total cholesterol excreted, we hypothesized that hypolipidemic activity of the extract may be mediated by reducing or inhibiting intestinal cholesterol absorption and increasing reverse cholesterol transport, as observed with agents inducing comparable hypolipidemic effects together with antioxidant effects such as Ezetimibe [29, 30] and bile acid sequestering cholestyramine [22, 31]. This reduction did not reach the normal rate after 4 weeks of treatment and the 4 weeks without treatment what allow us to say that the treatment during 4 weeks could be insufficient, but we noticed a reduction in the rate of VLDL-c during the 4 weeks without treatment, it could be that the extract of *C. occidentalis* continued to react and to show the effectiveness of the aqueous extract leaves. This effective action could be explained by the presence of the various chemical families present in the aqueous extract leaves. we limited our research to the treatment in 4 weeks without any time to reassure our self if after the treatment the formation of the atherom could continue and that the endothelium could find its integrity. The interest of this study consisted in checking that after 4 weeks of treatment, the extract would always act.the aim would be to highlight vasodilators which can reconstitute the level of integrity of the endothelium and the production and the diffusion of the oxide nitrite. The ratios TC/HDL and HDL/LDL are indexes of the coronary risk [32]. The ratios of atherogenicity TC/HDL and HDL/LDL of dyslipidemic rats treated with the extract of *C. occidentalis* were significantly reduced. These results reflect a lipidic profile antiatherogenic, and let suggest a protective effect of the extract with respect to the hypercholesterolemy induced by the mode enriched out of cholesterol. At the rats hyperlipidemic, saponins, steroid; especially saponins derived from the spirostanol, seem to be responsible for the reduction in total cholesterol. It was noted in all the cases a reduction in LDL-c and sometimes an increase in HDL-c. Saponins would act by formation of a complex with cholesterol or would have a direct effect on the metabolism of cholesterol. Several possible mechanisms of exercise-induced atheroprotective effects have been proposed such as increased HDL-c, decreased TC, and decreased oxidized LDL-c levels [33]. In the present study, endurance exercise and/or switching from the high fat to the normal diet improved lipid profiles by lowering the atherogenic plasma levels of total and LDL-c. However, the marked change in lipid profile observed concerned the plasma levels of the anti-atherogenic HDL-c. They were significantly increased in the exercise trained groups of rat (independently of the diet used) and not in the sedentary rats which were switched from the high fat to the control diet. Consequently, the atherogenic index was less in the exercise trained than in the rats with modified diet. Concerning plasma triglycerides, studies have also shown that they are strongly correlated with the prevalence and incidence of metabolic syndrome and cardiovascular diseases [34].

Many experimental studies showed the effectiveness of the medicinal plants or their extracts in the improvement of the activities of the enzymes implied in the metabolism of cholesterol [35]. Li et al. [36] showed that the increase in the activity of the Lecithin: Cholesterol Acyltransferase Activity (LCAT) and probably the reduction in that of hydroxy-methyl-glutary-coenzyme A reductase (HMG-CoA reductase), both ensuring the homeostasis of cholesterol, could be regarded as persons in charge for the reduction in the cholesterolemy, in the rat subjected to a feeding regime enriched with cholesterol treated with 5% by the extract by *Coriandrum sativum* during 75 days [36]. Moreover, Sudhop et al. [37] noted a rise in the activity of the LCAT and an inhibition of HMG-CoA reductase, in the rat made hypercholesterolemic treated with 500 mg/kg of an extract leaves of *Symplocos cochinchinensis*, during 28 days [38]. For this reason *C. occidentalis* could have an effect in the increase of the activity of the LCAT and in the reduction of hydroxy-methyl-glutary-coenzyme A reductase. it is very difficult to make a difference between efficacy of *C. occidentalis* with the efficacy of other plant extract without knowledge in the content of each plant extract. For the moment we explore the ways that *C. occidentalis* act and tried to explain the effect of the extract.

Our results showed in this model of hypercholesterolemy that several biomarkers of the oxydative stress faded in the rats subjected to the diet rich in cholesterol. The reduced glutathion is the most abundant endogenous antioxydant which interacts with activated oxygenated species, thus preventing the oxidation of the organic substrates (proteins, ADN, fatty acids). It is used as substrate of the glutathion peroxidase. The glutathion is also a trapper of radicals superoxydes and it protects the thiol groups from proteins against oxidation [39]. Our results showed a reduction in glutathione levels in the blood of the hypercholesterolemic rats. The determination of the specific activity of the superoxyde dismutase (SOD), enzyme which catalyses the dismutation of the anion superoxyde (O_2_^−^) out of water and hydrogen peroxide [40]. Faraci and Didion [40] revealed a decrease of the activity of the SOD in blood of the hypercholesterolemic rats. In response to the oxydative stress, the SOD is controlled in two different ways. In the event of moderate oxydative stress one observes a sur-expression of the SOD. If the oxydative stress persists the SOD is destroyed and its expression decreases. Paradoxically, an excessive concentration of the SOD can be dangerous because, in this case, it is the base of a hydrogen peroxide overproduction [41], would be secondary with the increase in the production of O_2_^−^ [42]., Malondialdehyde (MDA) is used as index of the lipidic peroxidation resulting from the reaction of the active species oxygenated with the membrane fatty acids [43]. In this study, the levels of MDA in blood was significantly increased in hypercholesterolemic rats. In treated group, the aqueous extract of *C. occidentalis* substantially prevented the decrease of GSH and the increase of MDA levels. The treatment also reduced the activity of the SOD. The polyphenols present in the aqueous extract of *C. occidentalis* [44], could also explain the antioxidant activity of this aqueous extract.

Gupta et al. [45], revealed that; treatment of rats with aqueous extract of *Annona squamosa* induced a decrease of VLDL-c, LDL, TG levels at the serum and liver and activity of HMG-CoA reductase. Moreover, this extract increased the rate of HDL-c, the activity of the LCAT and the synthesis of the biliary acids at the hepatic level, which involves a rise in the faecal excretion of cholesterol in rat subjected to a feeding regime enriched with cholesterol (2%) during 75 days. In the current study, it was observed a decrease in the TC, LDL-c, TG levels after treatment with *C. occidentalis* aqueous extract. However, the reduction of those parameters did not reach the normal and could be explained by the short treatment period (4 weeks).

The results of this study showed a significant increase in the faecal excretion of cholesterol, in the rats dyslipidemic or hyperlipidemic treated with aqueous extract of *C. occidentalis* compared with the untreated rats, which suggest an increase in the activity of the 7 α-hydroxylase, enzyme implicated in the transformation of cholesterol into biliary acids. These results are similar with those obtained with hypercholesterolemic rats, treated with aqueous extract of *Globularia alpum*, which observed an increase in the synthesis of the biliary acids at the hepatic level and an increase in faecal cholesterol [46].

The aqueous extract of *C. occidentalis* has an hypocholesterolemia effect and could then act effectively against the transport of cholesterol by the increase in the activity of the LCAT, thus resulting in enrichment of the HDL-c out of cholesterol esters.

## Conclusion

This study provides evidence of antihypercholesterolemia and hypotriglycemia effects of aqueous extract of *C. occidentalis.* It reduces Low Density Lipoprotein cholesterols (LDL-c), triglycerides (TG) and increasing High Density Lipoprotein cholesterols (HDL-c) in rats subjected to a feeding regime enriched with cholesterol. The diuretic activity of this extract justifies its use for the treatment of high blood pressure. However, toxicological studies need to be undertaken to ensure the safety use of this plant extract.

## Abbreviations

*C. occidentalis*: *Cassia occidentalis*
CAT: Catalase
dH_2_O: distilled water
GSH: Glutathion
HC: Hypercholesterolemic rats
HDL-c: High density lipoprotein cholesterol
HMG-CoA réductase: Hydroxy-methyl-glutary-coenzyme A réductase
LCAT: Lecithin: Cholesterol Acyltransferase Activity
LDL-c: Low density lipoprotein cholesterol
MDA: Malondialdehyde
NC: Normocholesterolemic rats
ROOH: Hydroperoxide
SOD: Superoxyde dismutase
T: Treatment
TC: Total cholesterol
TG: Triglyceride
VLDL-c: Very low density lipoprotein cholesterol
